# Bioactive Molecules against Rheumatoid Arthritis by Suppressing Pyroptosis

**DOI:** 10.3390/ph16070952

**Published:** 2023-07-03

**Authors:** Qian Zhou, Tian Li, Gang Fang, Yuzhou Pang, Xueni Wang

**Affiliations:** 1 Guangxi Zhuang Yao Medicine Center of Engineering and Technology, Guangxi University of Chinese Medicine, 13 Wuhe Road, Qingxiu District, Nanning 530200, China; 2 School of Basic Medical Science, Guangxi University of Chinese Medicine, 13 Wuhe Road, Qingxiu District, Nanning 530200, China; 3 School of Zhuang Medicine, Guangxi University of Chinese Medicine, 179 Mingxiudong Road, Xixiangtang District, Nanning 530001, China

**Keywords:** rheumatoid arthritis, pyroptosis, amiloride, paeoniflorin, punicalagin

## Abstract

Rheumatoid arthritis is an inflammatory disease, and pyroptosis is a form of death associated with an inflammatory response. Pyroptosis, which occurs in synovial and osteoblastic cells, can exacerbate the development of rheumatoid arthritis. The inhibition of pyroptosis of these cells can, therefore, clearly be used as a therapeutic strategy against rheumatoid arthritis. Here, we have summarized the current status of progress in the treatment of rheumatoid arthritis by targeting cellular pyroptosis. We have identified seven compounds, including a cyclic RNA, a microRNA, a peptide, and a cytokine (protein), that may influence the progression of rheumatoid arthritis by regulating the initiation of pyroptosis. All of these compounds have been shown to have anti-rheumatoid effects in vitro and/or in vivo and have the potential to be developed as anti-rheumatoid agents. These findings may help to accelerate the development of anti-rheumatoid arthritis drugs.

## 1. Introduction

Rheumatoid arthritis (RA) is the most prevalent and impactful chronic autoimmune rheumatologic disease in the world [1]. Approximately 1% of the general population worldwide is affected by RA [2,3]. RA is characterized by persistent synovial inflammation and irreversible damage to cartilage and bone [4,5]. It causes joint destruction and disability [3] and may also cause damage to multiple organs simultaneously, further limiting the patient’s physical, psychological, and social functioning [6]. Treat-to-target is currently the most widely used treatment strategy for patients with early RA. Treat-to-target therapy in the early stages of rheumatoid arthritis holds the promise of complete disease remission. However, if treatment fails, treat-to-target therapy can have many adverse effects, such as poor tolerability, low adherence, and safety issues for patients with rheumatoid arthritis caused by methotrexate, a drug commonly used in treat-to-target therapy [7]. Current drug therapies for the treatment of RA include nonsteroidal anti-inflammatory drugs, disease-modifying antirheumatic drugs, or biologic agents designed to reduce the symptoms of the disease, while there is currently no treatment available to cure RA [8,9]. Current clinical conventional drug therapy has limited therapeutic efficacy and is often associated with serious side effects, including hepatotoxicity; retinopathy; and cardiotoxicity disorders of the central nervous system, skin, blood, and infections [8,9,10]. Recent evidence suggests that novel targeted synthetic disease-modifying anti-rheumatic drugs, such as Janus-kinase (JAK) inhibitors, are superior to conventional therapy in combination with glucocorticoids for the treatment of early RA [11]. This approach may have advantages in terms of patient prognosis, but there are still concerns about serious adverse events that need to be addressed, such as atopic dermatitis [12], herpes zoster [13], and venous thromboembolism [14,15]. There is an urgent need to develop more effective and safer drugs to overcome RA.

Pyroptosis is a lytic and inflammatory programmed cell death, usually triggered by the inflammasome and executed by gasdermin (GSDM) proteins [16]. Upon recognition of exogenous or endogenous signals, cells undergo inflammasome assembly, cleavage of gasdermin, and release of pro-inflammatory cytokines and other cellular contents, ultimately leading to the death of inflammatory cells [17]. The onset of pyroptosis can be mediated by the caspase-1-dependent canonical pathway and the caspase-4/5/11-dependent non-canonical pathway [18]. During the initiation of pyroptosis, caspase-1 or caspase-11/4/5 is activated and cleaves gasdermin D (GSDMD) to separate its N-terminal pore-forming domain PFD. Oligomerization of the pore-forming domain forms macropores in the membrane, leading to cell swelling and membrane rupture [19]. The most striking difference between pyroptosis and other modes of cell death is that when pyroptosis is triggered, it rapidly causes cell swelling, membrane rupture, and lysis, resulting in the release of cell contents and large amounts of inflammatory factors and sending pro-inflammatory signals to neighboring cells, recruiting inflammatory cells and inducing an inflammatory response [18].

In recent years, studies have shown that inflammatory cytokines released by pyroptosis triggered by the caspase-1 pathway have the potential to induce the development of RA [20]. Pyroptosis releases IL-18 and IL-1β through the activation of inflammatory vesicles. IL-1β induces a procedure of inflammation, immune cell extravasation, and vasodilation that shapes immune responses that are adaptive in nature [21]. Pyroptosis not only proliferates and activates inflammatory bodies, thus triggering an intense inflammatory response, but also leads to activation of the immune system, especially T and B cells [22]. The activation of T and B cells is also an important pathological feature of RA. Therefore, pyroptosis may control the development of RA through the interaction of various intracellular kinases [20]. However, few studies have investigated the relationship between cellular pyroptosis and RA.

**Figure 1:**
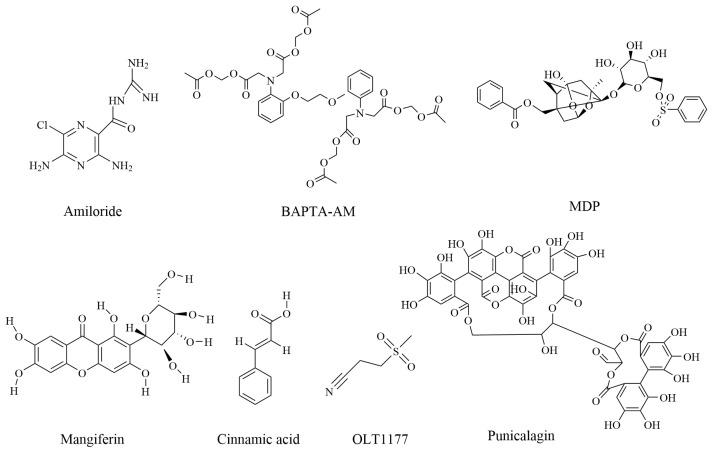
Chemical structure of compounds against rheumatoid arthritis by inhibition of pyroptosis.

This study identified seven compounds (Figure 1) (Table 1), two types of RNA (Table 2), one peptide, and one protein that can alleviate or treat RA by reducing the occurrence of osteoblast or synovial cell pyroptosis. The discovery of all these compounds provides new ideas for the treatment of RA. Mitigating or inhibiting the development of RA by regulating the onset of cell death through the mechanism of pyroptosis may become a strategy that receives increasing attention.

## 2. Compounds against Rheumatoid Arthritis by Suppressing Pyroptosis

### 2.1. Amiloride

Amiloride is a potent potassium-sparing diuretic with natriuretic and antipotassium properties [23]. Amiloride is neuroprotective in a model of cerebral ischemia, a property attributable to the drug’s inhibition of central ASIC [23]. Amiloride is a non-selective acid-sensitive ion channel (ASIC) blocker that inhibits acid-induced pain. ASICs are cation channels that are activated by extracellular acidosis [24]. Extracellular acidosis significantly increases the expression of acid-sensitive ion channel 1a (ASIC1a), IL-1β, IL-18, ASC, NLRP3, and caspase-1 and promotes the release of lactate dehydrogenase. ASIC1a is a member of the degenerin/epithelial sodium channel protein superfamily, which is transiently activated by extracellular H+ and plays a key role in a variety of physiological and pathological processes, including RA [24]. ASIC1a is a unique subunit that transports Ca^2+^, whereas other ASICs only affect Na+ [25]. ASIC1a has been shown to be present in rat articular chondrocytes, and the upregulation of ASIC1a expression in arthritic rats leads to articular chondrocyte apoptosis [26].

Studies have shown that amiloride reduces ASIC1a-induced articular chondrocyte apoptosis and pro-inflammatory cytokine release [27]. In the experiment by Wu et al., they found that amiloride not only reduced ASIC1a-induced articular chondrocyte apoptosis but also reduced ASIC1a-induced articular chondrocyte pyroptosis [28]. In detail, amiloride- treated adjuvant arthritis rats morphologically improved the pathological joint changes of arthritis (including synovial hyperplasia, synovial thickening, pannus formation, and infiltration of a variety of inflammatory cells). Furthermore, amiloride inhibited the degeneration of Col 2a Agg in articular cartilage and thus had a protective effect on RA patients. In addition, they also demonstrated that amiloride can inhibit the overexpression of ASIC1a, ASC, NLRP3, caspase-1 gene, and pro-inflammatory cytokines IL-1β and IL-18 in articular chondrocytes by Western blotting, PCR, and Elisa assay. In conclusion, Wu demonstrated that amiloride can inhibit pyroptosis of articular chondrocytes through the ASIC1a pathway.

### 2.2. BAPTA-AM

1,2-bis-(o-aminophenoxy)-ethane- N,N,N′,N′--tetraacetic acid tetra-acetoxymethyl ester (BAPTA-AM) is an intracellular calcium chelator [29]. Wu et al. found that BAPTA-AM reduced hypoxia–reoxygenation-induced caspase-1-GSDMD processing in hepatic macrophages by reducing calcium levels, thereby reducing hepatic macrophage pyroptosis and decreasing the levels of IL-1β, IL-18, and lactate dehydrogenase [30]. Wu investigated the changes in Ca^2+^ content in articular chondrocytes when ASIC1a-mediated extracellular acidosis occurred [28]. Confocal micrographs confirmed that extracellular acidosis could significantly increase the Ca^2+^ content in articular chondrocytes. In addition, BAPTA-AM could inhibit the levels of IL-1β and IL-18 and the mRNA expression of ASC, NLRP3, caspase-1, and IL-1β in articular chondrocytes by decreasing the Ca^2+^ content [28].

The above experimental results demonstrated that the upregulation of Ca^2+^ in chondrocytes leads to the expression, aggregation, and assembly of NLRP3 inflammasomes, followed by the activation of caspase-1 and, finally, chondrocyte pyroptosis. In addition, they verified the same conclusion in animal experiments. Therefore, ASIC1a may mediate extracellular acidosis-induced articular chondrocyte pyroptosis through the regulation of Ca^2+^. BAPTA-AM could attenuate extracellular acidosis-induced pyroptosis in primary articular chondrocytes based on the expression of ASC, NLRP3, and cleaved caspase-1p10 [28].

### 2.3. Mangiferin and Cinnamic Acid

Baihu-Guizhi decoction is an anti-rheumatic formula of Chinese herbs with good clinical efficacy in the treatment of RA [31]. Baihu-Guizhi decoction is composed of Gypsum Fibrosum, Anemarrhenae Rhizoma, Cinnamomi Ramulus, Oryza sativa, and Glycyrrhizae Radix et Rhizoma, as recorded by Chinese medical sage Zhongjing Zhang in *The Synopsis of Prescriptions of the Golden Chamber.* Mangiferin (MG) is a natural compound that could be purified from Anemarrhenae Rhizoma, and cinnamic acid (CA) is a natural molecule from Cinnamomi Ramulus. MG and CA were identified as potential bioactive compounds in Baihu-Guizhi decoction by Li through an integrated research strategy combining UFLC-Q-TOF-MS/MS, gene expression profiling, network calculation, pharmacokinetic profiling, surface plasmon resonance, microscale thermophoresis, and pharmacological experiments [32].

On this basis, Li conducted in vitro and in vivo experiments to determine the drug effects and pharmacological mechanisms [32]. Combined treatment with MG and CA reversed histopathologic changes in the knee joint of adjuvant-induced arthritis-modified rat model (AIA-M) rats, including reduced inflammatory cell infiltration, attenuated cartilage and bone destruction, and synovial hyperplasia. The combined treatment of MG and CA may be effective in attenuating the progression of bone damage and repairing bone erosion. The combined treatment of MG and CA protected the morphological structure of the spleen and thymus of mice, while significantly reducing the spleen and thymus indices of mice. The increased expression of NLRP3, ASC, caspase-1, IL-1b, and IL-18 in the AIA-M rat arthritis model group was reversed by the combined treatment of MG and CA. The N-terminal domain of GSDMD activity in serum and the level of the N-terminal domain of GSDMD in the knee joint were significantly increased in AIA-M rats, and the N-terminal domain of GSDMD activity was significantly decreased after combined treatment with MG and CA. In addition, the combined treatment of MG and CA also demonstrated inhibition of TLR4/PI3K/AKT/nuclear factor-κB (NF-κB) signaling activation in AIA-M rat sera. Notably, a series of cellular experiments using LPS/ATP-induced RAW264.7 and MH7A cells also confirmed the above results. In conclusion, the combination of MG and CA alleviates joint inflammation and bone erosion by inhibiting NF-kB via TLR4/PI3K/AKT signaling to suppress NLRP3 inflammasome activation, leading to the downregulation of downstream caspase-1, the reduced release of IL-1b and IL-18, and modulation of GSDMD-mediated pyroptosis [32].

### 2.4. OLT1177

OLT1177 is a sulfonyl cyanide compound that targets the NLRP3 inflammasome, thereby blocking the processing and release of IL-1β and IL-18 in vitro. OLT1177 can reduce the severity of endotoxin-induced inflammation in vivo [33]. In Marchetti’s study, OLT1177 had anti-inflammatory effects in mouse models of reactive arthritis and gouty arthritis, respectively [34]. They observed a significant reduction in the levels of inflammatory cytokines, including IL-1β and IL-6, in synovial tissue explants, and OLT1177 treatment inhibited the infiltration of cells into the joint, as evidenced by a reduction in the levels of the neutrophil chemokine CXCL1 in synovial tissue extracts. They next examined the effect of OLT1177 on NLRP3 inflammasome formation using immunofluorescence and fluorescence resonance energy transfer analysis. Fluorescence resonance energy transfer analysis confirmed that LPS/nigericin treatment results in the formation of NLRP3-ASC and NLRP3-(pro) caspase-1 complexes at a distance of less than 30 nm, which was significantly reduced in the presence of OLT1177 [33].

In addition, they confirmed the inhibition of NLRP3-ASC oligomerization by OLT1177 using an immunoprecipitation assay. Crucially, they demonstrated that OLT1177 dose-dependently reduced the severity of zymosan-induced arthritis in mice by intraperitoneal administration and by oral gavage. At the same time, the anti-inflammatory properties of OLT1177 treatment in the zymosan or monosodium urate crystal-induced arthritis model were also confirmed by the reduction of phospho-c-Jun N-terminal kinase phosphorylation [34]. Phospho-c-Jun N-terminal kinase has been implicated in the pathophysiology of several forms of arthritis, such as RA and gouty arthritis [34].

### 2.5. The Monomeric Derivatives of Paeoniflorin

The most obvious feature of RA is synovitis, and synovial tissue hypoxia is one of the important pathological features of synovitis. Under hypoxia, the level of reactive oxygen species (ROS) increased significantly in fibroblast-like synoviocytes due to a wide range of changes in mitochondrial structure, dynamics, and genome stability [35]. The massive accumulation of ROS led to the massive accumulation of hypoxia-inducible factor-1α (HIF-1α) [36,37,38]. At present, some studies have found that HIF-1α is an important cause of cell pyroptosis [39].

Paeoniflorin, the main active constituent of *Paeonia lactiflora*, has been shown to prevent CoCl2- and hypoxia-induced HIF-1α accumulation and downregulate the expressions of p53 and Bcl-2/adenovirus E1B 19 kDa interacting protein 3, thereby affecting apoptosis [40]. However, the bioavailability of paeoniflorin is not ideal due to its highly hydrophilic nature [41]. The monomeric derivatives of paeoniflorin (MDP) developed by Hong showed superior bioavailability and efficacy over paeoniflorin [41].

In Hong’s research [41], Western blot analysis showed that HIF-1α, GSDMD-N, NLRP3 inflammatory vesicles, and cleaved-caspase-1 were highly expressed in the fibroblast-like synoviocytes of RA patients. Interestingly, they were restored after treatment with MDP. Flow cytometry showed an increased expression of HIF-1α and NLRP3 inflammatory vesicle mRNA levels in the nucleus of fibroblast-like synoviocytes under hypoxic conditions. Hypoxia-induced pyroptosis of fibroblast-like synoviocytes was significantly attenuated when ROS, HIF-1α, GRK2, or NLRP3 expression was eliminated. Hong found that HIF-1α could induce pyroptosis in fibroblast-like synoviocytes by activating NLRP3 inflammatory vesicles. Hong found that MDP could reduce HIF-1α expression through the targeted inhibition of G protein-coupled receptor kinase 2S670 phosphorylation and GRK2 in fibroblast-like synoviocytes, thereby preventing NLRP3 inflammatory vesicle activation, which in turn inhibited fibroblast-like synoviocyte pyroptosis and alleviated synovial inflammation.

In Hong’s animal experiments [41], MDP inhibited the high expression of HIF-1α and GSDMD-N in adjuvant arthritis rats and attenuated the inflammatory response in adjuvant arthritis rats. In conclusion, hypoxia induces pyroptosis in fibroblast-like synoviocytes by activating the ROS/GRK2/HIF-1α/NLRP3 pathway, and MDP could reduce hypoxia-induced pyroptosis in fibroblast-like synoviocytes by inhibiting the GRK2/HIF-1α axis. In this regard, MDP has great potential to be clinically translated as a therapeutic agent for the treatment of RA. In addition, MDP has also been shown to affect the pyroptosis process of other cells. MDP inhibits macrophage pyroptosis by inhibiting TLR4 in vivo and in vitro [41].

### 2.6. Punicalagin

Punicalagin, one of the major active compounds in pomegranate peel, has been reported to possess many bioactivities, including antioxidant, antimicrobial, antiviral, and immunosuppressive activities [42]. Ge et al. [43] found that punicalagin can inhibit the progression of RA in a variety of ways. Punicalagin could partially alleviate arthritis by inhibiting the pro-inflammatory effects of macrophages.

Activated macrophages exhibit two distinct phenotypes: the M1 (classical) phenotype and the M2 (alternative) phenotype [44]. Macrophages play a key role in the pathogenesis of RA [45]. During the development of RA, macrophages are mainly polarized toward the M1 phenotype and release proinflammatory cytokines, including inducible nitric oxide synthase iNOS, tumor necrosis factor (TNF)-α, IL-1β, and IL-6 [46]. M2 macrophages act as inflammation suppressors by releasing anti-inflammatory cytokines, facilitating apoptotic neutrophil phagocytosis, and inhibiting damaging immune system activation by cytokines such as IL-10 [47].

In Ge’s experiments [43], the immunofluorescence staining of inducible nitric oxide synthase and Arg-1 in the synovium showed that punicalagin treatment converted macrophages from the M1 to M2 phenotype after lipopolysaccharide and interferon (IFN)-γ stimulation. Punicalagin reduced the levels of synovial pro-inflammatory factors. In addition, punicalagin attenuated pyroptosis by downregulating NLRP3 and caspase-1 expression, thereby preventing inflammatory cell death caused by IL-1β and IL-18 release. Mechanistically, punicalagin inhibited the activation of receptor activators of the NF-κB signaling pathway, which contributes to M1 polarization and pyroptosis of macrophages. In addition, punicalagin could directly inhibit the formation of osteoclasts induced by the receptor activator of nuclear factor-κB ligand, which further verified the bone protective effect of punicalagin. Punicalagin was found to inhibit bone destruction in CIA mice in vivo and to reduce inflammation in vivo and in vitro. Animal studies have also shown that punicalagin not only improves arthritis symptoms and subchondral bone fractures but also reduces systemic bone loss without toxic effects on the liver or kidneys.

**Table 1:** The influenced proteins of action of compounds.

No	Compounds	Influenced Proteins	References
1	Amiloride	ASIC1a, NLRP3	[28]
2	BAPTA-AM	ASIC1a, NLRP3	[28]
3	Mangiferin	NF-κB, NLRP3	[32]
4	Cinnamic acid	NF-κB, NLRP3	[32]
5	OLT1177	NLRP3	[33,34]
6	The monomeric derivatives of paeoniflorin	HIF-1a, NLRP3	[41]
7	Punicalagin	NLRP3	[43]

## 3. RNA, Peptide, and Protein against Rheumatoid Arthritis by Modulating Pyroptosis

### 3.1. Hsa_circ_0044235

Circular RNAs are recently discovered endogenous non-coding RNAs with a covalently closed circular structure [48]. Hsa_circ_0044235 has been shown to be significantly downregulated in RA patients [49]. Sirtuin 1 protein (SIRT1) is closely associated with the pyroptosis pathway. When SIRT1 is downregulated, it inhibits cell proliferation and activates cell pyroptosis [50]. Silencing SIRT1 reduces the proliferation and potential adhesion of fibroblast-like synoviocytes, suggesting that SIRT1 is also an important molecular marker in RA [51].

Chen’s experiments [52] showed that Hsa_circ_0044235 and SIRT1 expression was suppressed and positively correlated in RA patients. The overexpression of hsa_circ_0044235 promoted the expression of SIRT1 and significantly inhibited the expression of NLRP3, ASC, cleaved caspase-1, and IL-1β. The above results suggest that has_circ_0044235 may affect the NLRP3 inflammatory vesicle-mediated pyroptosis pathway through the indirect regulation of SIRT1. By obtaining overlaps in multiple databases, Chen found that miR-135b-5p could specifically target SIRT1. In addition, the effect of overexpressed hsa_circ_0044235 on the pyroptosis pathway was reversed by miR-135b-5p mimic, while the promotion of caspase-1, NLRP3, ASC, and IL-1β expressions by siSIRT1 was reversed by miR-135b-5p inhibitor. Furthermore, miR-135b-5p expression was increased in RA patients and LPS/ATP-induced fibroblast-like synoviocytes. The overexpression of has_-circ_0044235 partially reduced miR-135b-5p expression in the LPS/ATP-induced cell pyroptosis pathway in fibroblast-like synoviocytes. In addition, the miR-135b-5p inhibitor significantly reduced the effect of siSIRT1 in promoting NLRP3 activation. The above results demonstrate that Hsa_circ_0044235 regulates the development of RA through the regulation of NLRP3-mediated fibroblast-like synoviocyte pyroptosis by the miR-135b-5p-SIRT1 axis. In animal studies, the overexpression of hsa_circ_0044235 reduced joint inflammation, apoptosis, and joint damage; decreased paw pad thickness; and reduced clinical case scores in CIA mice.

### 3.2. miR-144-3p

The miR-144-3p expression was upregulated in IL-1b-induced N1511 mouse chondrocytes [53]. The expression of NLRP3, cleaved caspase-1, GSDMD, and cleaved caspase-3 in N1511 mouse chondrocytes was also significantly upregulated by IL-1b, but the upregulation of all these proteins could be reversed by miR-144-3p knockdown. This finding suggests that miR-144-3p knockdown could reduce chondrocyte pyroptosis in N1511 mice caused by IL-1b. In other words, miR-144-3p knockdown can attenuate the progression of RA by reducing chondrocyte pyroptosis.

### 3.3. Psalmotoxin 1

Psalmotoxin 1, a peptide purified from the venom of the southern spider tarantula Psalmopoeus cambridgei, can specifically and strongly inhibit the current of ASIC1a [54]. Wu [28] found that the high expression of ASIC1a, NLRP3 inflammatory vesicle component, IL-1β, IL-18, and positive AO/EB staining upregulated Lactate dehydrogenase expression after ph6.0 stimulation of articular chondrocytes. Interestingly, the ASIC1a-specific inhibitor Psalmotoxin 1 reversed the effects of extracellular acidosis on articular chondrocytes. In addition to this, Psalmotoxin 1 reduced the expression levels of ASC and caspase-1 in articular chondrocytes. Wu suggested that Psalmotoxin 1 could potentially attenuate extracellular acidosis-induced cellular pyroptosis in articular chondrocytes by regulating NLRP3.

Xu [55] and Wu [27,28] found that in animal experiments, the HE staining and toluidine blue staining of relevant cells receiving Psalmotoxin 1 joint injections showed improved synovial invasion and cartilage destruction, reduced joint swelling, and significantly less severe arthritis compared to untreated adjuvant arthritis rats. In addition, Psalmotoxin 1 reduced extracellular acidification-induced invasion and migration of RA-fibroblast-like cells, as well as the expression of MMP2, MMP9, and p-FAK that were upregulated by ASIC1a [56]. In contrast, ASIC1a overexpression increased synovial inflammation and invasion [56,57]. This suggests that ASIC1a may be a major regulator of synovial infiltration and joint inflammation. In addition, another study mentioned that Psalmotoxin 1 inhibits the increase of the acid-induced necrosis marker RIP1, suggesting that ASIC1a can also mediate acid-induced chondrocyte necrosis [58]. Therefore, blocking ASIC1a-mediated chondrocyte pyroptosis or necrosis may also provide a potential therapeutic strategy for the treatment of RA.

### 3.4. IL-37

IL-37 is an anti-inflammatory cytokine that was first reported in 2000 [59,60]. It has been reported to be elevated in RA synovial fluid, serum, and plasma [61,62]. Bulau et al. observed that mature IL-37 translocates to the nucleus and inhibits the expression of pro-inflammatory cytokines associated with pyroptosis, such as caspase-1 and NLRP3 [63,64]. Ren’s study showed that IL-37 attenuated TNF-α-induced pyroptosis in RA FLSs by inhibiting the NF-κB/GSDMD signaling pathway [65]. Furthermore, IL-37 could reduce the severity of arthritis in CIA rats [65]. Based on the above findings, IL-37 is potentially useful for the treatment of RA.

**Table 2:** The sequence of RNA and Peptide.

No	RNA or Peptide	Base Sequence (5′–3′)	Reference
1	hsa_circ_0044235	TGAGTTTGGTGATTCAGCTTGC, AACAAGGCTTCTTCTGAGTGT	[52]
2	SIRT1	TGATTGGCACCGATCCTCG, CCACAGCGTCATATCATCCAG	[52]
3	miR-144-3p	ucauguagUAGAUAUGACAu	[53]
4	Psalmotoxin 1	EDCIPKWKGCVNRHGDCCEGLECWKRRRSFEVCVPKTPKT (Modifications: Disulfide bonds: 3–18, 10–23, 17–33)	[56]

## 4. Perspective

During the development of RA, the accumulation of intra-articular inflammatory metabolites leads to a decrease in joint fluid pH, resulting in extracellular acidosis and the activation of ASIC1a [24], which promotes Ca^2+^ influx into cells. The upregulation of intercellular Ca^2+^ contributes to the expression, aggregation, and assembly of NLRP3 inflammatory vesicles, which subsequently activates caspase-1 and cleaves Il-1β and Il-18 to their biologically active forms [30]. This ultimately leads to pyroptosis of articular chondrocytes, resulting in the erosion and destruction of articular cartilage and bone. In the present study, we found that amiloride, BAPTA-AM, and psalmotoxin 1 inhibited the overexpression of ASIC1a, ASC, NLRP3, caspase-1 gene, and pro-inflammatory cytokines IL-1β and IL-18 in articular chondrocytes. Common to all three compounds is that they all affect the onset of cellular pyroptosis in articular chondrocytes via the ASIC1a pathway. Additionally, ASIC1a may mediate extracellular acidosis-induced articular chondrocyte pyroptosis through the regulation of Ca^2+^. ASIC1a may be a potential therapeutic target for RA. We expect that it will be possible to develop effective RA drugs that target pyroptosis and IL-1β or IL-18 through the ASIC1a pathway.

Synovial hypoxia is also an important factor in the development of RA [66,67]. Fibroblast-like synoviocytes induce the overproduction of ROS through mitochondrial damage under hypoxic conditions, which increases the synthesis of HIF-1α [68,69]. The high accumulation of ROS leads to an increase in GRK2 levels. More importantly, the phosphorylation of GRK2 regulates HIF-1α. NLRP3 inflammatory vesicle expression is mediated by HIF-1α under hypoxic conditions [70]. NLRP3 inflammatory vesicles can recruit and activate caspase-1 via the adaptor molecule ASC, and this activated caspase-1 can cleave GSDMD, IL-1β, and IL-18 to their mature forms, which subsequently trigger cellular pyroptosis [71]. Several studies have now shown that HIF-1α is an important cause of cellular pyroptosis [72]. In RA, HIF-1α is highly expressed in synovial tissue and contributes significantly to inflammatory gene expression and cell survival in the synovium [73]. Therefore, there is an urgent need for an effective therapeutic strategy for RA that inhibits HIF-1α. MDP can attenuate hypoxia-induced fibroblast-like synovial cell pyroptosis by inhibiting the GRK2/HIF-1α axis. Thus, MDP has great clinical potential for the treatment of RA. Punicalagin can improve pathological inflammation through multiple pathways without toxic effects on the liver or kidneys. Punicalagin has low toxicity and may be administered by intra-articular injection in the future for the treatment of RA. Punicalagin has great potential for the treatment of RA. However, the absorption efficiency, actual effect, and appropriate dose of punicalagin in the joint cavity need to be further investigated. We found that collected bioactive molecules regulate cell pyroptosis by activating NLRP3 inflammasomes and then activating the caspase-1 pathway, thereby affecting the process of RA. The difference is that the upstream factors that cause the activation of NLRP3 inflammasomes are different. We expect that, in the future, other upstream factors or compounds will be discovered that can directly target NLRP3 inflammasomes.

Clinically, anti-inflammatory drugs and disease-modifying antirheumatic drugs have been widely used to treat patients with RA [74]. However, the adverse effects of these drugs, such as gastrointestinal reactions, abnormal changes in liver function, and bone marrow suppression, make it impossible to achieve long-term disease remission [75].

**Figure 2:**
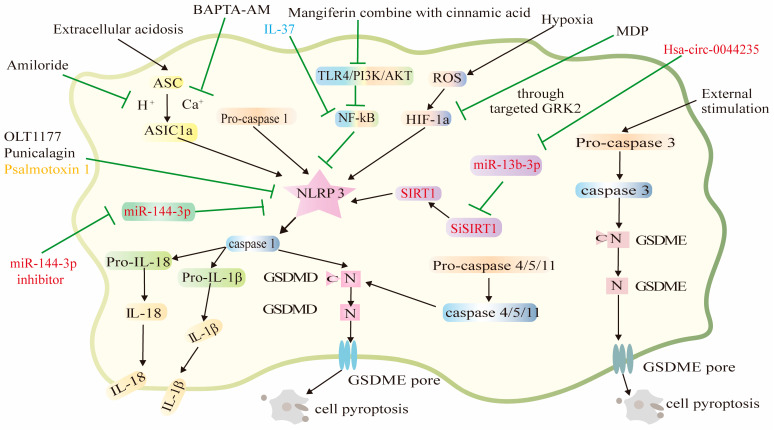
Overview of the mode of action of biomolecules in the regulation of pyroptosis (ASIC1a, acid-sensitive ion channel 1a; BAPTA-AM, 1,2-bis-(o-aminophenoxy)-ethane- N,N,N′,N′--tetraacetic acid, tetra-acetoxymethyl ester; HIF-1α, hypoxia-inducible factor-1α; MDP, the monomeric derivatives of paeoniflorin; NF-κB, nuclear factor-Κb; ROS, reactive oxygen species; SIRT1, Sirtuin 1 protein).

In addition, surgical treatment is usually applied in the advanced stage of RA, but these operations are traumatic for the patient and cannot achieve a satisfactory result. Therefore, there is an urgent need for new methods to effectively treat RA. The compounds, RNA, peptide, and protein identified in this paper can be used to treat RA by pyroptosis (Figure 2), and in particular, the use of punicalagin does not have toxic effects on the liver or kidneys. This is because the activation of the immune system by pyroptosis allows the immune cells to specifically recognize the target cells, greatly reducing the toxic effects of the drug on normal tissues. Some compounds may even protect RA patients during treatment. However, it will be a long time before these compounds become clinical agents for the treatment of RA. If these drugs, based on pyroptosis mechanisms for the treatment of RA, can be administered in precisely controlled doses, the safety of the drugs will be largely assured. Pyroptosis can positively enhance the body’s immune response. It is expected that pyroptosis may provide a new, less harmful, and better treatment for RA.

Pyroptosis is an innate immune response that fights infection and plays an important role in the normal functioning of the immune system [76]. However, pyroptosis is a double-edged sword [16,77,78,79]. Appropriate inflammatory response and induction of pyroptosis can enhance pathogen clearance. Pyroptosis also leads to the activation of the immune system, particularly helper T cell 1 (Th1) and helper T cell 2 (Th2) [80]. However, excessive pyroptosis can lead to a hyperinflammatory response and exacerbate tissue damage, which can lead to inflammatory diseases and immune disorders. Therefore, excessive pyroptosis should be given high priority in disease management. RA is a common and frequent disease, and although the clinical treatment for RA is abundant, no specific drugs for RA treatment have been found, and there is also a lack of systematic and comprehensive treatment to achieve the level of a cure for the disease. In addition, the high disability rate of RA seriously affects the quality of life of patients. Pyroptosis is a novel form of cell death, but the relationship between pyroptosis and RA is poorly understood. The recent discovery of compounds that interfere with the progression of RA through the cell pyroptosis pathway has opened our eyes to new ideas and new approaches for the treatment of RA. However, how to regulate the appropriate cell pyroptosis to achieve the desired therapeutic effect and how to avoid normal cell pyroptosis damage during the treatment of RA remain to be explored. Among other things, the specific mechanisms of how different compounds regulate cell pyroptosis should be explored. Although much is still unknown about the regulatory role of cell pyroptosis in RA, an in-depth study of the mechanisms of cell pyroptosis regulation may help to better exploit the potential of cell pyroptosis in the treatment of RA. We hope that the information collected in this paper can provide new ideas for experimental research and the clinical use of drugs.

## Data Availability

Not applicable.
